# Isolation, Characterization, and Functional Properties of Antioxidant Peptides from Mulberry Leaf Enzymatic Hydrolysates

**DOI:** 10.3390/antiox13070854

**Published:** 2024-07-16

**Authors:** Yichen Zhou, Rijun Zhang, Junyong Wang, Yucui Tong, Jing Zhang, Zhenzhen Li, Haosen Zhang, Zaheer Abbas, Dayong Si, Xubiao Wei

**Affiliations:** Laboratory of Feed Biotechnology, State Key Laboratory of Animal Nutrition and Feeding, College of Animal Science and Technology, China Agricultural University, Beijing 100193, China; yichenzhou51@cau.edu.cn (Y.Z.);

**Keywords:** mulberry leaf, antioxidant peptide, oxidative damage, apoptosis, free radical scavenging

## Abstract

Recent evidence suggests that mulberry leaves have good antioxidant activity. However, what the antioxidant ingredient is and how the ingredient works are still not well understood. In this study, we enzymatically hydrolyze mulberry leaf proteins (MLPs) using neutral protease and find that the mulberry leaf protein hydrolysates (MLPHs) have stronger antioxidant activity compared to MLPs. We separate the core antioxidant components in MLPHs by ion-exchange columns and molecular sieves and identify 798 antioxidant peptides by LC-MS/MS. Through bioinformatics analysis and biochemical assays, we screen two previously unreported peptides, P6 and P7, with excellent antioxidant activities. P6 and P7 not only significantly reduce ROS in cells but also improve the activities of the antioxidant enzymes SOD and CAT. In addition, both peptides are found to exert protective effects against H_2_O_2_-induced chromatin damage and cell apoptosis. Collectively, these results provide support for the application of mulberry leaf peptides as antioxidants in the medical, food and livestock industries.

## 1. Introduction

At the basal level in organisms, reactive oxygen species (ROS), such as hydroxyl radicals, hydrogen peroxide, and superoxide anion, can act as signaling molecules and growth factors in physiological and biochemical processes in the body [1,2]. However, excessive accumulation of ROS in the body disrupts redox homeostasis, leading to oxidative damage to cell membranes, lipids, proteins, and DNA [3]. This imbalance can induce a variety of diseases, including diabetes, cancer, and cardiovascular disorders [4,5]. To protect themselves from ROS-induced toxicity, cells have evolved a complex antioxidant defense system consisting of enzymatic antioxidants, such as catalase (CAT), superoxide dismutase (SOD), and glutathione peroxidase (GSH-Px) [6]. However, when the level of oxidative stress exceeds the body’s own regulatory limits, it is necessary to regulate it with exogenous antioxidant reagents. Natural antioxidants are favored for their minimal side effects, environmental friendliness, and safety and have significant advantages over their synthetic counterparts [7,8].

China is the world’s largest producer of mulberry leaves, but a large amount is wasted every year. Recent studies have shown that mulberry leaves have excellent antioxidant and immunomodulatory properties [9,10], suggesting a great potential for clinical, food, and livestock development applications. Although many antioxidant components, such as flavonoids, polyphenols, and polysaccharides, have been identified in mulberry leaves [11,12], whether mulberry leaves contain antioxidant peptides and how they work still remains largely unclear. The in vitro enzymatic digestion of proteins is an important method for obtaining bioactive peptides. Compared with proteins, peptides have the advantages of low molecular weight, easy absorption, and good stability [13]. Mulberry leaves are rich in crude protein and have an excellent amino acid composition, which provides the possibility of the enzymatic digestion of mulberry leaves to obtain antioxidant peptides.

In this study, we investigated whether antioxidant peptides can be obtained by the enzymatic hydrolysis of mulberry leaf proteins and preliminarily explored their antioxidant activities and the underlying mechanisms. These findings will provide insight into the antioxidative functions of mulberry leaves, expand our understanding of mulberry leaf antioxidant peptides, and suggest new opportunities for clinical, food, and animal husbandry antioxidant applications.

## 2. Materials and Methods

### 2.1. Materials and Chemicals

Mulberry leaves (Yue Sang 69851), harvested in June, were obtained from the Mulberry Orchard Farm in Long’an County, Guangxi, China. The 100 Da and 3.5 kDa dialysis bags were purchased from QiMeng Biotechnology Co., Ltd. (Changsha, China). 

2,2′-Azino-bis (3-ethylbenzothiazoline-6-sulfonic acid) diammonium salt (ABTS), penicillin–streptomycin solution (PS), JC-1, Hoechst 33258, the bicinchoninic acid (BCA) protein concentration assay kit, 2′,7′-dichlorofluorescin diacetate (DCFH-DA), and 6-hydroxy-2,5,7,8 tetramethylchroman-2-carboxylicacid (Trolox) were provided by Solarbio Science & Technology Co., Ltd. (Beijing, China). Neutral protease was purchased from Aobox Biotechnology Co., Ltd. (Beijing, China). The Cell Counting Kit-8 (CCK-8) assay kit was obtained from Biosharp Technology Co., Ltd. (Hefei, China). 1,1-Diphenyl-2-picrylhydrazyl (DPPH) was acquired from TCI Development Co., Ltd. (Tokyo, Japan). Dulbecco’s Modified Eagle Medium (DMEM) and phosphate-buffered solution (PBS) powder were acquired from Thermo Fisher Scientific Inc. Fetal bovine serum (FBS) was purchased from Wuhan Procell Life Technology Co., Ltd. (Wuhan, China). The Annexin V-FITC apoptosis detection kit was obtained from Nanjing Nest Biotechnology Co., Ltd. (Nanjing, China). SOD and CAT assay kits were supplied by Jiancheng Biotechnology Co. Ltd. (Nanjing, China). H_2_O_2_ was purchased from Sigma-Aldrich. Sephadex G-25 and DEAE Sepharose FF were obtained from Beijing Ruida Henghui Technology Development Co., Ltd. (Beijing, China). 

### 2.2. Preparation of Mulberry Leaf Protein (MLP) and Mulberry Leaf Protein Hydrolysates (MLPHs)

Fresh mulberry leaves were thoroughly washed and air-dried overnight. Subsequently, they were dried to a constant weight in an oven at 55 °C, ventilated and cooled, and then ground in a crusher. The mulberry leaf powder obtained after sieving through a 60-mesh sieve was stored at −20 °C.

The MLP and MLPHs were prepared following the procedure described in a previous study [14]. In detail, mulberry leaf powder was mixed with deionized water, and the pH was adjusted to 11, followed by sonication (235 w, 4.9 min) at 30 °C and, finally, extraction. The supernatant was collected post-centrifugation (6000× *g*, 10 min), and then the pH was adjusted to 4.0; the supernatant was allowed to stand for 20 min and re-centrifuged (8000× *g*, 10 min). The supernatant was discarded, and the precipitate was washed twice with deionized water. After redissolving and neutralizing, desalting was conducted in a 3.5 kDa cut-off dialysis bag for 48 h. The desalted MLP extract was stored at −20 °C. 

MLP was dissolved at 1% (*w*/*v*) in deionized water and heat-treated at 90 °C to deactivate protease inhibitors. Neutral proteases were then used to digest MLP under optimal conditions (pH = 7.0, 45 °C, 6000 U/g enzyme addition). After digestion, proteases were inactivated by boiling for 10 min. The mixture was cooled and centrifuged (5000× *g*, 10 min) to separate the components. The supernatant was collected and dialyzed using a 100 Da cut-off bag for 48 h. The resulting MLPHs were freeze-dried and stored at −20 °C.

### 2.3. Separation of Antioxidant Components of MLPHs

The DEAE Sepharose FF matrix was loaded into a glass column (2.6 cm × 20 cm) and equilibrated. A 10 mg/mL MLPH solution was prepared with Tris-HCl (20 mM, pH = 7.8) and filtered through a 0.45 μm membrane. The MLPH solution (10 mL) was added to the column and washed with NaCl-Tris-HCl gradients (0 to 1.0 M) at 1 mL/min. Absorbance at 220 nm was monitored to plot the elution curve. The peaks were collected, desalted in 100 Da bags, concentrated by rotary evaporation, and then tested for DPPH radical scavenging. The components with the strongest scavenging ability were identified for further purification.

The Sephadex G-25 matrix was packed into a 2.6 cm × 20 cm glass column and equilibrated. Fractions F3, F4, and F5 (the components with the strongest scavenging ability in the last experiment) were prepared at a concentration of 5 mg/mL in distilled water and filtered through a 0.45 μm membrane, and 2 mL of each was loaded onto the column. Elution was conducted with distilled water at 0.5 mL/min, collecting one tube every 6 min for 60 tubes. Absorbance at 220 nm was measured to plot the elution curve. The peak components were collected, and their DPPH free radical scavenging activity was assessed. The most active components were selected for peptide sequence identification.

### 2.4. Identification of Bioactive Mulberry Peptides (BMPs) by HPLC-MS

The sample solution was prepared with DTT at a concentration of 10 mM and reacted in a 56 °C water bath for 1 h. IAM was added to reach a final concentration of 55 mM and reacted in the dark for 40 min. Subsequently, the solvent was desalted using a self-packed desalting column and dried in a vacuum centrifugal concentrator at 45 °C.

After drying, the samples were resuspended in 50 μL of 0.1% formic acid and then analyzed by HPLC-MS. The chromatography system was EASY-nLC 1200 UPLC, and the mass spectrometer was Q Exactive™ Hybrid Quadrupole-Orbitrap™ Mass Spectrometer. 

The sample was injected into an Acclaim PepMap RPLC C18 column (1.9 μm, 100 Å, 150 μm × 150 mm) at a flow rate of 600 nL/min. Mobile phase A was 0.1% formic acid, and mobile phase B was an 80% ACN/0.1% formic acid solution. The elution conditions were as follows: 0–2 min, 4% B to 8% B; 2–45 min, 8% B to 28% B; 45–55 min, 28% B to 40% B; 55–56 min, 40% B to 95% B; 56–66 min, 95% B. The mass spectrometry scanning range was set to 300–1800 *m*/*z*, and the raw mass spectrometry files were analyzed using PEAKS Studio 10.6 with the de novo sequencing method for peptide sequence analysis.

### 2.5. In Silico Analysis of Physicochemical Properties of BMPs

We performed an in silico analysis of the physicochemical properties of BMPs. We analyzed and screened the peptides based on their hydrophobicity (https://pepdraw.com/ (accessed on 15 June 2023)), bioactivity score (http://distilldeep.ucd.ie/PeptideRanker/ (accessed on 15 June 2023)), toxicity (https://webs.iiitd.edu.in/raghava/toxinpred/design.php (accessed on 15 June 2023)), and solubility (https://pepcalc.com/ (accessed on 15 June 2023)). The selected peptides were synthesized (≥98% purity) by GL Biochem (Shanghai, China). 

### 2.6. Determination of ABTS, DPPH, and OH Scavenging Capacities

First, 300 μL of the sample solution was mixed with 2 mL of the ABTS working solution (OD_734_ = 0.7 ± 0.02). OD_734_ was measured after 10 min of reaction at room temperature, protected from light. The ABTS free radical scavenging rate was calculated using the following formula:

$$ABTS\;Free\;radical\;scavenging\;rate(\%)=\frac{\left(1-({OD}_{Sample}-{OD}_{Blank}\right)}{{OD}_{Control}}\times 100\%$$

Next, 1 mL of the sample solution was mixed with 2 mL of the DPPH working solution (0.2 mM, dissolved in anhydrous ethanol). OD_517_ was measured after 20 min of reaction at room temperature, protected from light. The DPPH free radical scavenging rate was calculated using the following formula:

$$DPPH\;Free\;radical\;scavengingrate(\%)=\frac{\left(1-({OD}_{Sample}-{OD}_{Blank}\right)}{{OD}_{Control}}\times 100\%$$

Finally, 1 mL of the sample solution was mixed with 1.5 mL of salicylic acid (1.8 mM), 2 mL of ferric sulfate (1.8 mM), and 1 mL of hydrogen peroxide (6 mM). After incubation at 37 °C for 30 min, the reaction mixture was centrifuged at 3000× *g* for 5 min. The supernatant was collected, and the OD_510_ was measured. The OH free radical scavenging rate was calculated using the following formula:

$$OH\;Free\;radical\;scavenging\;rate(\%)=\frac{\left(1-({OD}_{Sample}-{OD}_{Blank}\right)}{{OD}_{Control}}\times 100\%$$

### 2.7. Determination of Reducing Power

First, 1 mL of the sample solution was mixed with 2.5 mL of sodium carbonate buffer (0.2 M, pH = 6.6) and 2.5 mL of potassium ferricyanide solution (1%, *w*/*v*). After a 20 min reaction at 50 °C, 2.5 mL of trichloroacetic acid solution (10%, *w*/*v*) was added. After centrifugation (3000× *g*, 10 min), 2.5 mL of the supernatant was taken and mixed with an equal volume of deionized water and 0.5 mL of 0.1% (*w*/*v*) FeCl_3_ solution. After a 10 min incubation at room temperature, the OD_600_ was measured.

### 2.8. Cell Culture and Cell Viability Assay

HepG2 cells were cultured in a complete medium (90% DMEM + 10% FBS + 100 units/mL penicillin and 100 μg/mL streptomycin) at 37 °C, 5% CO_2_, and 90% relative humidity. The Cell Counting Kit-8 (CCK-8) Assay Kit was used to detect the effect of H_2_O_2_ and peptides on cell viability. HepG2 cells (1 × 10^5^ cells/mL) were pre-seeded in a 96-well culture plate overnight and then treated with H_2_O_2_ and peptides at 37 °C for 24 h. Cell viability was assessed with the CCK-8 kit (Item No. BS350C) according to the protocol supplied by the manufacturer. 

### 2.9. The Protective Effect of BMPs against H_2_O_2_-Induced Oxidative Damage in HepG2 Cells

We first established a H_2_O_2_-induced oxidative damage model in HepG2 cells. HepG2 cells (1 × 10^5^ cells/mL) in a volume of 100 μL were seeded in a 96-well culture plate and incubated at 37 °C for 24 h. The control group and damage group received 100 μL of DMEM, the positive control group received 100 μL of 100 μg/mL Trolox, and the experimental group received 100 μL of DMEM containing different concentrations of peptides. 

After culturing for another 6 h, the experimental and damage groups were exposed to H_2_O_2_ (160 μM) for 24 h, while the control group received an equal volume of DMEM. Finally, cell viability was measured following the procedures in Section 2.8.

### 2.10. Determination of SOD and CAT Activities in HepG2 Cells

First, HepG2 cells (1 × 10^5^ cells/mL) in a volume of 2 mL were seeded in a 6-well culture plate and incubated at 37 °C for 24 h. Experimental grouping and treatments were the same as in Section 2.9. Next, cells were collected, and cell lysis was performed using RIPA cell lysis solution. The protein concentration in cell lysates was determined by the BCA protein assay kit. SOD (Item No. A001-3-2) and CAT (Item No. A007-1-1) activity assays were carried out according to the instructions provided with the corresponding assay kits.

### 2.11. Determination of ROS Levels in HepG2 Cells

HepG2 cells were treated as in Section 2.10. After that, the supernatant was discarded, and cells were gently washed twice with PBS. Subsequently, the cells were incubated with DMEM containing 10 μM DCFH2-DA at 37 °C for 20 min. After the incubation, the cells were carefully washed twice with PBS, and then 1 mL of DMEM was added, after which the cells were observed under a fluorescence microscope. 

The contents of ROS were indicated by DCF fluorescence and quantified using excitation and emission filters of 488 and 525 nm, respectively. The ROS levels were expressed as a percentage of the blank control values.

### 2.12. Hoechst 33258 and JC-1 Staining

HepG2 cells were treated as in Section 2.10. Hoechst 33258 (Item No. C0021) and JC-1 (Item No. M8650) staining was performed according to the instructions provided in the staining kit. 

After JC-1 staining, the samples were examined under a fluorescence microscope. JC-1 aggregates and monomers showed green fluorescence (excitation at 485 nm/emission at 535 nm) and red fluorescence (excitation at 530 nm/emission at 590 nm), respectively. The red/green fluorescence ratio can be considered a direct indicator of the state of mitochondrial polarization.

Hoechst 33258 staining was directly observed using a fluorescence microscope. 

### 2.13. Determination of Apoptosis Rate of HepG2 Cells

HepG2 cells were treated as in Section 2.10. After that, cells were collected, rinsed twice with PBS, and then resuspended in Apoptosis Detection Staining solution (containing 2 μL of Annexin V-FITC dye, 5 μL of PI dye, and 100 μL of Binding buffer). 

After 15 min of dark incubation at room temperature, cells were analyzed for their apoptotic status (Ex = 488 nm, FL1 EM = 515 nm, FL2 EM = 560 nm) using the BD Fortessa flow cytometer (Becton Dickinson, Franklin Lakes, NJ, USA), and the data were analyzed with Flowjo software 10.0. 

### 2.14. Statistical Analysis

Statistical analyses were performed using GraphPad Prism 9.4.1 software (GraphPad Software, Inc., San Diego, CA, USA). *p* values were calculated using one-way ANOVA. Data are means ± Standard Deviations (SDs). NS: *p* > 0.05; *: *p* ≤ 0.05; **: *p* ≤ 0.01; and ****: *p* ≤ 0.0001.

## 3. Results

### 3.1. Antioxidant Capacity of MLP and MLPHs

**Figure 1:**
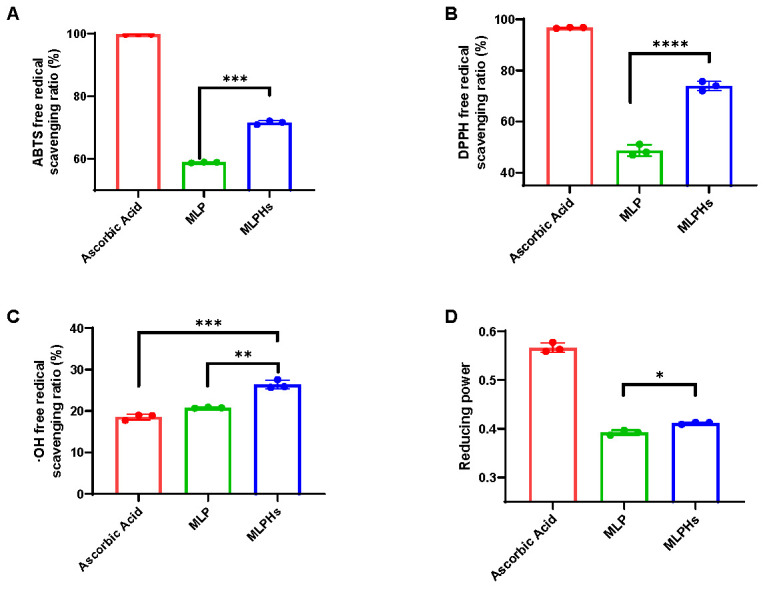
Antioxidant capacities of mulberry leaf protein (MLP) and mulberry leaf protein hydrolysates (MLPHs). (**A**) ABTS free radical scavenging abilities of ascorbic acid (0.1 mg/mL), MLP (0.1 mg/mL), and MLPHs (0.1 mg/mL). (**B**) DPPH scavenging activities of ascorbic acid (0.1 mg/mL), MLP (0.1 mg/mL), and MLPHs (0.1 mg/mL). (**C**) OH free radical scavenging activities of ascorbic acid (0.1 mg/mL), MLP (0.1 mg/mL), and MLPHs (0.1 mg/mL). (**D**) Reducing power of ascorbic acid (0.1 mg/mL), MLP (0.1 mg/mL), and MLPHs (0.1 mg/mL). Data are representative of three independent biological replicates. Data are means ± SDs. *: *p* ≤ 0.05; **: *p* ≤ 0.01; ***: *p* ≤ 0.001; and ****: *p* ≤ 0.0001.

The ability of the samples to scavenge ABTS, DPPH, and OH radicals can reflect their antioxidant capacity [15]. We found that MLP had strong antioxidant activity, with a rate of OH radical scavenging comparable to that of ascorbic acid (Figure 1C). MLPHs showed stronger antioxidant activity in all four antioxidant assays compared to MLP (Figure 1), especially ABTS (Figure 1A) and DPPH radical scavenging capacities (Figure 1B). Furthermore, the OH radical scavenging rate of MLPHs was as high as 26.39 ± 1.05% at a concentration of 0.1 mg/mL, which was significantly higher than that of ascorbic acid (*p* < 0.001). 

The antioxidant activity of MLPHs was significantly higher than that of MLP, which, to some extent, demonstrated that some hydrolysis components with antioxidant activity were produced after enzymatic digestion. Therefore, we conducted further experiments to elucidate what the antioxidant products are.

### 3.2. Isolation and Characterization of BMPs from MLPHs

**Figure 2:**
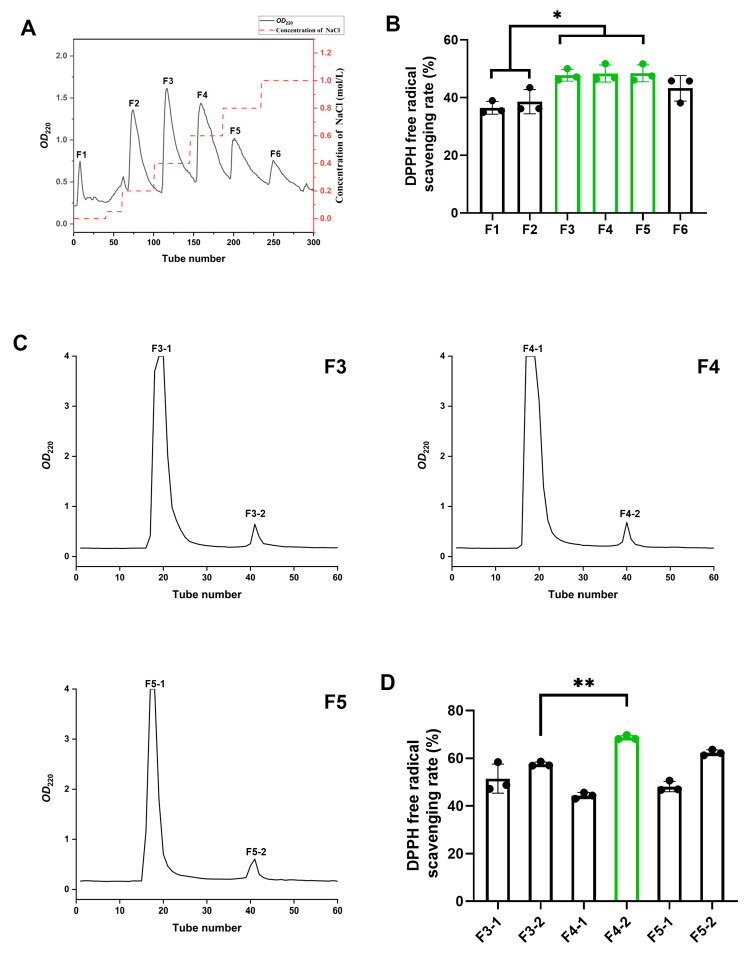

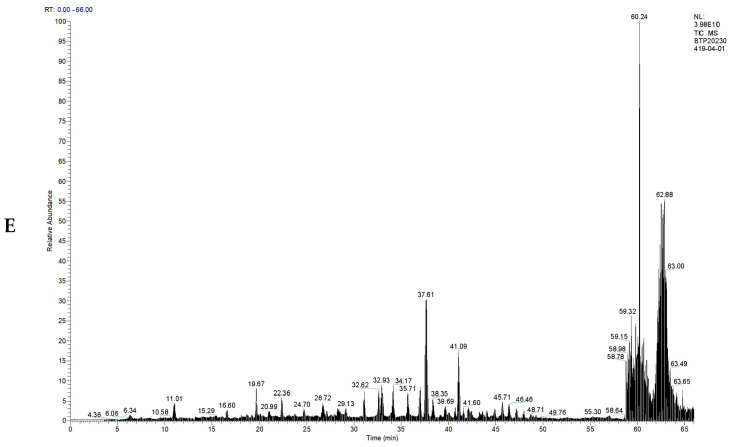
The isolation and characterization of bioactive mulberry peptides (BMPs) from mulberry leaf protein hydrolysates (MLPHs). (**A**) The separation chromatogram of MLPHs by DEAE Sepharose FF. F1-F6 are the six major isolates of MLPHs. (**B**) The DPPH radical scavenging activity of the F1-F6 fractions mentioned in (**A**). (**C**) The separation chromatogram of F3, F4, and F5 by Sephadex G-25. F3, F4, and F5 were all separated into 2 major components, which were exhibited as two peaks (F3-1, F3-2; F4-1, F4-2; F5-1, F5-2). (**D**) The DPPH radical scavenging activity of fractions F3-1, F3-2, F4-1, F4-2, F5-1, and F5-2 mentioned in (**C**). (**E**) The total ion chromatogram of F4-2. Data are representative of three independent biological replicates. Data are means ± SDs. *: *p* ≤ 0.05 and **: *p* ≤ 0.01.

We speculated that the MLPHs in Figure 1 exhibit significantly higher antioxidant activity than the MLP due to the enzymatic production of new antioxidant peptides. Due to the polarity of amino acid side chains, different proteins and peptides carry different types and amounts of charge in polar liquid media. Based on this property, the MLPHs were subjected to a DEAE Sepharose FF ion-exchange column to separate components with different charges. MLPHs were separated into six main components (Figure 2A). Based on the peak areas, F2, F3, and F4 are the main components of MLPHs (Figure 2A). We further determined and compared the antioxidant capacity of these six ingredients through the DPPH radical scavenging assay (Figure 2B). The results indicated that the DPPH radical scavenging rates of components F3, F4, and F5 were significantly higher than those of the other components (Figure 2B), so we selected these three components for further separation.

We used Sephadex G-25 to further separate peptides with different molecular weights in these three components (Figure 2C). F3, F4, and F5 were each divided into two components, shown as a major peak and a minor peak (Figure 2C). We named them F3-1, F3-2, F4-1, F4-2, F5-1, and F5-2. It showed that the DPPH radical scavenging rates of peptide peaks with lower molecular weights (F3-2, F4-2, F5-2) were higher than those of peptide peaks with higher molecular weights (F3-1, F4-1, F5-1) (Figure 2D). Since the antioxidant capacity of component F4-2 was significantly higher than that of other components, we selected F4-2 for further experiments.

HPLC-MS was employed to determine the sequences of peptides in the F4-2 fraction. The total ion chromatogram of F4-2 is shown in Figure 2E. A peptide sequence analysis was performed using the de novo sequencing method in PEAKS Studio10.6, and a total of 798 peptide sequences were identified, of which 45 peptides had a confidence score ≥95 (Supplementary Information Figure S1). 

Based on PepRanks prediction, 39% of the identified peptides may have biological activity, with peptide lengths concentrated between 6 and 12 amino acids (Supplementary Information Figure S2). 

### 3.3. Virtual Screening of BMPs 

The physicochemical properties of the 45 peptides mentioned in Figure S1 were predicted using the online database mentioned in Section 2.5. We ranked the peptides based on their predicted biological activity scores using the PeptideRankers database. Afterward, we predicted the toxicity and water solubility of the top eight peptides, which were named P1, P2, P3, P4, P5, P6, P7, and P8 (Table 1). The amino acid sequences of these eight peptides are shown in Table 2. These peptides were synthesized (≥ 98% purity) (Figure S3) by GL Biochem (Shanghai, China) for further study. 

### 3.4. Screening of BMPs with Antioxidant Activity by Cellular Assay

**Figure 3:**
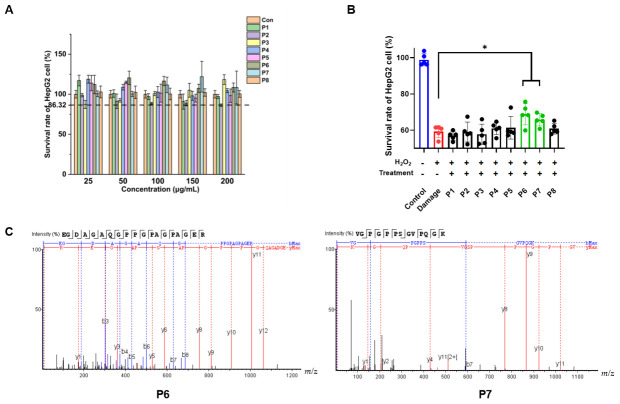
The screening of bioactive mulberry peptides (BMPs) with antioxidant activity by a cellular assay. (**A**) The effects of BMPs on the survival rate of HepG2 cells. HepG2 cells were treated with P1-P8 (25–200 μg/mL) for 24 h, and the survival rate was determined by CCK8. (**B**) The protective effects of P1-P8 against H_2_O_2_-induced damage in HepG2 cells. HepG2 cells were pretreated with P1-P8 (100 μg/mL) for 6 h and then treated with H_2_O_2_ (160 μM) for 24 h. The cell survival rate was determined by CCK8. (**C**) Secondary mass spectra of P6 and P7. Data are representative of three independent biological replicates. Data are means ± SDs. *: *p* ≤ 0.05.

In the concentration range of 50–200 μg/mL, no cytotoxicity of P1-P8 was observed, suggesting that this concentration range of P1-P8 is suitable for the following experiments (Figure 3A). H_2_O_2_ permeates cell membranes and generates free radicals like O^2-^· and OH· through a series of biochemical reactions, inducing cell damage and death [16]. The impact of H_2_O_2_ on cell viability is depicted in Figure S3. After treating the cells with 160 μM H_2_O_2_ for 24 h, the cell survival rate was close to 50%, and we finally chose this concentration to establish the H_2_O_2_-induced oxidative stress model. We investigated the mitigating effect of peptides on H_2_O_2_-induced oxidative damage in HepG2 cells (Figure 3B). The effects of P6 and P7 were significantly better than those of the other peptides (Figure 3B), so we chose these two peptides for the follow-up study.

### 3.5. P6 and P7 Reduce H_2_O_2_-Induced ROS Accumulation and Oxidative Damage 

**Figure 4:**
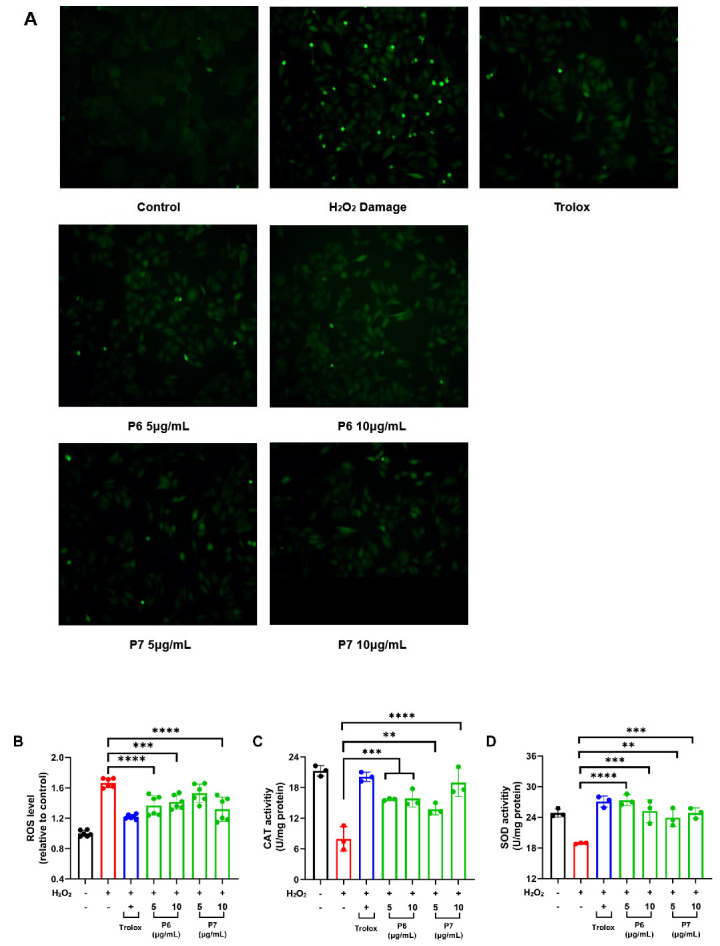
The effects of P6 and P7 on ROS, CAT, and SOD levels in HepG2 cells. (**A**) HepG2 cells were pretreated with Trolox (0.1 mg/mL), P6 (5–10 μg/mL), or P7 (5–10 μg/mL) for 6 h and then treated with H_2_O_2_ (160 μM) for 24 h. The cells were then stained with DCFH-DA and imaged by fluorescence microscopy (200×). (**B**) The fluorescence intensity in (**A**) was determined by a microplate reader. (**C**) The effects of P6 and P7 on CAT activity in HepG2 cells. Cells were treated as in (**A**). (**D**) The effects of P6 and P7 on SOD activity in HepG2 cells. Cells were treated as in (A). Data are representative of three independent biological replicates. Data are means ± SDs. **: *p* ≤ 0.01; ***: *p* ≤ 0.001; and ****: *p* ≤ 0.0001.

Excessive ROS accumulation leads to oxidative damage in cells [17]. DCFH-DA staining is a commonly used method to measure the level of intracellular ROS. DCFH-DA can freely penetrate the cell membrane, and after deacetylation by esterase in the cell, it will be converted to DCFH, which can no longer penetrate the cell membrane. The non-fluorescent DCFH is easily oxidized by intracellular ROS to form the highly fluorescent 2′,7′-dichlorofluorescein (DCF) and can therefore be used to characterize the levels of intracellular ROS [18]. As expected, the fluorescence intensity of H_2_O_2_-treated cells was very high (Figure 4A,B). However, the cellular fluorescence intensity was significantly reduced by the addition of P6 and P7 (*p* < 0.01), indicating that P6 and P7 could effectively reduce H_2_O_2_-induced ROS accumulation and oxidative damage (Figure 4A,B).

The antioxidant enzymes SOD and CAT play a vital role in the body’s defense against oxidative damage and can directly eliminate ROS. The role of SOD is to catalyze the superoxide anion and convert it to hydrogen peroxide to reduce cellular toxicity, while CAT directly breaks down hydrogen peroxide and prevents the production of toxic hydroxyl radicals. Therefore, we further evaluated changes in the SOD and CAT enzymes. H_2_O_2_ caused a significant decrease in the levels of CAT and SOD in HepG2 cells, whereas treatment with P6 and P7 resulted in a significant increase in the activities of SOD and CAT enzymes (*p* < 0.01) (Figure 4C,D). Combining the above results, P6 and P7 could alleviate H_2_O_2_-induced oxidative damage by increasing the activities of CAT and SOD.

### 3.6. Protective Effect of P6 and P7 against Chromatin Damage

**Figure 5:**
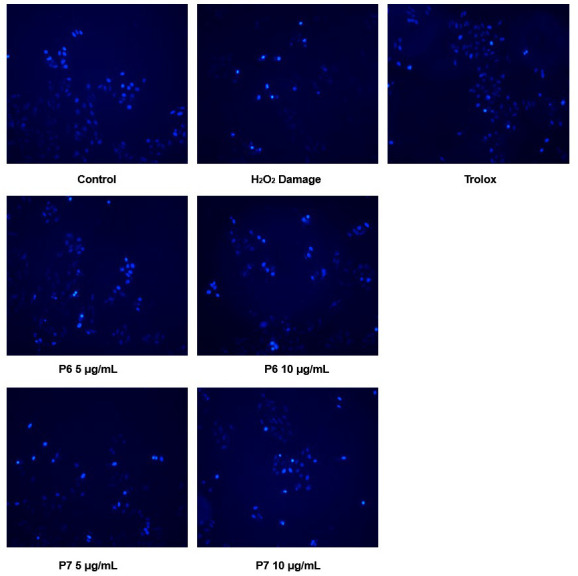
The effects of P6 and P7 on chromatin damage in HepG2 cells. HepG2 cells were pretreated with Trolox (0.1 mg/mL), P6 (5–10 μg/mL), or P7 (5–10 μg/mL) for 6 h and then treated with H_2_O_2_ (160 μM) for 24 h. The cells were then stained with Hoechst 33258 and imaged by fluorescence microscopy (200×).

Oxidative damage is usually accompanied by chromatin damage. We therefore further investigated the protective effects of P6 and P7 against H_2_O_2_-induced DNA damage using Hoechst 33258 staining. Nuclear staining of normal cells (control group) showed uniform, faint blue fluorescence, whereas nuclear staining of chromatin-damaged cells (H_2_O_2_ damage group) showed condensed, fragmented, and bright blue fluorescence (Figure 5). Cells pretreated with P6 and P7 displayed reduced blue fluorescence and fewer bright spots, suggesting their protective effect against DNA oxidative damage.

### 3.7. Protective Effect of P6 and P7 against Cell Apoptosis

Oxidative damage can induce cell apoptosis, and a decrease in mitochondrial membrane potential is an important marker of apoptosis [19]. JC-1 staining enables the rapid and sensitive detection of changes in cellular mitochondrial membrane potential. When the mitochondrial membrane potential is high, JC-1 aggregates in the mitochondrial matrix and produces red fluorescence. In contrast, when the mitochondrial membrane potential is low, JC-1 exists as a monomer and cannot aggregate in the mitochondrial matrix, emitting green fluorescence. Therefore, the transition of JC-1 fluorescence from red to green can be used as an early indicator of cell apoptosis [20].

**Figure 6:**
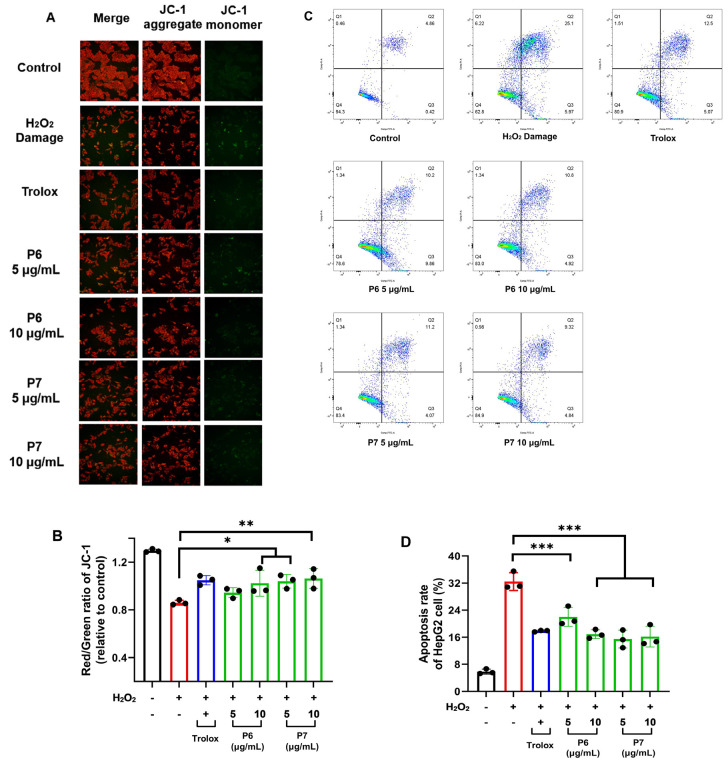
The protective effect of P6 and P7 against cell apoptosis. (**A**) HepG2 cells were pretreated with Trolox (0.1 mg/mL), P6 (5–10 μg/mL), and P7 (5–10 μg/mL) for 6 h and then treated with H_2_O_2_ (160 μM) for 24 h. The cells were then stained with JC-1 and imaged by fluorescence microscopy (200×). (**B**) The fluorescence intensity in (**A**) was determined by a microplate reader. Cells were treated as in (**A**). (**C**) The detection of apoptosis by flow cytometry using the Annexin V–FITC assay. Cells were treated as in (**A**). (**D**) The effects of P6 and P7 on the apoptosis rate in HepG2 cells. Cells were treated as in (**A**). Data are representative of three independent biological replicates. Data are means ± SDs. *: *p* ≤ 0.05; **: *p* ≤ 0.01; ***: *p* ≤ 0.001.

H_2_O_2_ caused a significant increase in the green fluorescence of HepG2 cells with a round and condensed morphology, indicating a decrease in mitochondrial membrane potential and the occurrence of cell apoptosis. However, pretreatment with P6 and P7 reduced green fluorescence (Figure 6A). Furthermore, the red/green fluorescence ratio of cells in the H_2_O_2_ damage group was significantly lower compared to the control group (*p* < 0.01), which was effectively alleviated by P6 and P7 (Figure 6B). This demonstrated the protective effect of P6 and P7 against cell apoptosis.

Phosphatidylserine (PS) flipping to the outer membrane and membrane rupture are important markers of early and late apoptosis, respectively. Annexin-V can bind to flipped PS, and PI can penetrate damaged cell membranes to bind to intracellular nucleic acids. Therefore, Annexin-V–FITC/PI staining can be used to detect the apoptosis rate of cells. Annexin-V–FITC/PI staining assay indicated that P6 and P7 significantly reduced the apoptosis rate of HepG2 cells.

## 4. Discussion

In this study, we first discovered that MLP exhibits enhanced antioxidant activity after being hydrolyzed by neutral protease. This suggested that the enzymatic hydrolysis of proteins leads to changes in antioxidant capacity, which is consistent with a previous study that found that the antioxidant properties of hempseed proteins were enhanced after alkaline hydrolysis and bromelain hydrolysis [21]. We speculated that this phenomenon may be due to the generation of antioxidant peptides during the enzymatic hydrolysis process. Consequently, we further separated and purified the MLPHs and found that peptides with lower molecular weights exhibited stronger antioxidant activity during the separation process, which is consistent with previous studies [22,23]. Studies have suggested that antioxidant peptides always have lower molecular weights, possibly due to their simpler structures and exposure of hydrophobic R groups [24]. Additionally, studies have suggested that due to the fact that peptides with molecular weights between 500 and 1500 Da are more readily absorbed by intestinal epithelial cells, they could exhibit better antioxidant activity [23,25,26]. Although the molecular weight is crucial for the antioxidant activity of peptides, it is particularly important to note that, in addition, amino acid composition, peptide sequence, conformation, and hydrophobicity also have a significant impact on the antioxidant activity of peptides [27]. In this part of the study, due to the extreme complexity of MLPHs, multi-step separation was required to minimize the range of antioxidant components, but it should be noted that the final concentration of the separated sample should not fall below the peptide detection threshold. This pair of contradictions greatly increased the difficulty of the experiment. In this experiment, we performed only a two-step separation of MLPHs, which simplified the separation process but also led to a huge number of peptides in the final fraction, posing a challenge for subsequent screening. Balancing the convenience of separation and the accuracy of identification deserves further study. To facilitate the subsequent experiments more conveniently, this study selected the PeptideRanker database for the preliminary screening of active peptides, which has been used in other similar studies [28]. For example, Montone utilized LC-MS/MS and the PeptideRanker database to identify 500 peptides from Tetradesmus obliquus microalgae, of which four short peptides (WPRGYFL, GPDRPKFLGPF, WYGPDRPKFL, and SDWDRF) were found to have particularly prominent antioxidant activity [29].

Subsequently, we opted for cell-based assays with the aim of more accurately screening antioxidant peptides and assessing their efficacy. In this study, we finally found that two antioxidant peptides, P6 and P7, could reduce the ROS levels by increasing the activity of intracellular antioxidant enzymes, thereby alleviating oxidative damage to mitochondrial membrane and chromatin and ultimately decreasing H_2_O_2_-induced cell apoptosis. It should be emphasized that the primary objective of this study is to obtain antioxidant peptides from mulberry leaves and preliminarily verify their antioxidant activity; hence, the exploration of the underlying mechanisms is relatively superficial. For instance, the question of how peptides P6 and P7 enhance the activity of intracellular antioxidant enzymes—whether it is at the level of gene transcription or protein translation or modification—also warrants further investigation. Apart from that, some other mechanisms of antioxidant peptides have also been reported. It has been shown that the Keap1/Nrf2 signaling pathway plays a crucial role in regulating the expression of intracellular antioxidant enzymes. Yin found that two peptides (APDPFRMY and NGGPDWAR) obtained from the enzymatic hydrolysis of tilapia skin proteins possessed antioxidant capacity and demonstrated that they were able to inhibit Keap1-mediated Nrf2 degradation and promote the expression of downstream antioxidant genes [22]. Hence, further studies will be required to characterize more in-depth molecular mechanisms of P6 and P7.

## 5. Conclusions

We performed enzymatic digestion of MLP and isolated and identified two peptides with excellent antioxidant activity, P6 and P7, from the enzymatic hydrolysates. P6 and P7 not only reduce ROS in cells but also improve the activities of the antioxidant enzymes SOD and CAT. In addition, both peptides were found to exert protective effects against H_2_O_2_-induced chromatin damage and cell apoptosis. These findings provide theoretical and technical support for the utilization of mulberry leaf resources and the development of P6 and P7 as novel natural antioxidants. In addition, our study contributes to the growing body of research on antioxidant peptides and expands our understanding of them.

## Figures and Tables

**Table 1:** Eight BMPs obtained by virtual screening and their physicochemical properties.

Peptide	Score	Bioactivity	Solubility	Toxicity	Length	*m/z*	Z	RT	Mass	Ppm
P1	97	0.861147	good	Non-Toxin	11	507.7345	2	25.69	1013.445	8.9
P2	95	0.860728	good	Non-Toxin	9	390.2128	2	6.95	778.4085	3.3
P3	99	0.833139	good	Non-Toxin	9	434.7379	2	10.68	867.4562	5.7
P4	99	0.819956	good	Non-Toxin	9	418.7236	2	7.52	835.43	3.2
P5	95	0.803338	good	Non-Toxin	7	321.1724	2	5.85	640.3292	1.5
P6	99	0.795404	good	Non-Toxin	11	448.2379	2	7.09	894.4559	6
P7	95	0.744693	good	Non-Toxin	19	845.8934	2	14.89	1689.771	1.1
P8	97	0.725901	good	Non-Toxin	13	588.8252	2	14.9	1175.641	−4.5

**Table 2:** Sequences of P1–P8.

Peptide	Sequence
P1	GPAGPAGPR
P2	GSPPGEGPAGF
P3	GPSGPQGLR
P4	SGPAGPR
P5	GPAGPSGAPGK
P6	EGDAGAQGPPGPAGPAGER
P7	RPGPSPGVGAPGK
P8	GPAGPQGPR

## Data Availability

Data will be made available on request.

## Supplementary Materials

The following supporting information can be downloaded at https://www.mdpi.com/article/10.3390/antiox13070854/s1: Figure S1: Peptide identification results (confidence score ≥95); Figure S2: Predicted results of bioactivity (A) and length distribution (B) of identified mulberry leaf peptide; Figure S3: Effects of different concentrations of H_2_O_2_ on the survival rate of HepG2 cells.
