# Supplementary material for: Isolation, Characterization, and Functional Properties of Antioxidant Peptides from Mulberry Leaf Enzymatic Hydrolysates
Source: Antioxidants (Basel). 2024 Jul 16;13(7):854. doi: 10.3390/antiox13070854 (PMC11273431; doi:10.3390/antiox13070854)
Supplement: Supplementary file 1 [file antioxidants-13-00854-s001.zip › antioxidants-3041087-supplementary.pdf]

1	Peptide	Scan	Score	length	m/z	z	RT	Area	Mass	ppm
2	GPAGPQGPR	1847	99	9	418.7236	2	7.52	3.78E+07	835.43	3.2
3	GPTGPAGVR	2598	99	9	406.2263	2	9.82	1.34E+07	810.4348	4
4	GSEGPQGVR	1934	99	9	443.7244	2	7.86	8.26E+06	885.4304	4.3
5	GPAGPSGAPGK	1758	99	11	448.2379	2	7.09	8.01E+06	894.4559	6
6	Q(+42.01)AGPQGPR	1576	99	8	426.7201	2	6.39	6.64E+06	851.4249	0.8
7	GPSGPQGLR	3066	99	9	434.7379	2	10.68	3.09E+06	867.4562	5.7
8	Q(+42.01)AGPAGPK	1320	99	8	384.2062	2	6.08	2.97E+06	766.3973	0.6
9	DGAGGPVGR	1683	99	9	393.1993	2	7.03	2.48E+06	784.3828	1.7
10	DDPSQSANLLAEAK	8085	99	14	729.8561	2	27.04	2.22E+06	1457.6997	-1.4
11	VAMGPR	4148	99	6	315.6732	2	14.14	1.20E+06	629.3319	-0.2
12	LEAC(+57.02)VKAR	2353	99	8	473.7633	2	9.14	1.15E+05	945.5065	5.9
13	VATVSLPR	3834	99	8	421.7643	2	13.13	0	841.5021	14.2
14	LHSANVL	3837	99	7	377.2204	2	13.13	0	752.4181	10.8
15	AEFVEVTK	3815	99	8	461.7555	2	13.09	0	921.4807	17.1
16	Q(+42.01)VGPSPGVGPAGK	4036	98	13	596.8223	2	13.8	2.30E+07	1191.6248	4.4
17	A(+42.01)GPAGAVAAGATQR	4709	98	14	620.3278	2	15.16	1.32E+07	1238.6367	3.5
18	QPPSGDGQPGAK	1671	98	12	569.7812	2	7.27	1.03E+07	1137.5415	5.7
19	A(+42.01)GRGPSGPAGA	1639	98	11	470.2375	2	6.84	8.94E+05	938.457	3.6
20	KLNDAQAPK	1617	98	9	492.779	2	6.83	7.55E+05	983.54	3.5
21	QPAGRP	1334	98	6	313.1745	2	6.08	2.72E+05	624.3344	0.1
22	GETGPAGPAGPVGVPGAR	6426	97	18	773.9014	2	21.01	1.02E+08	1545.7898	-1
23	RPGPSPGVGAPGK	4742	97	13	588.8252	2	14.9	1.39E+07	1175.6411	-4.5
24	A(+42.01)GADGVAATKW	6071	97	11	544.7744	2	19.74	1.10E+07	1087.5298	4.1
25	VGSPGPAQR	1787	97	9	434.7369	2	7.46	6.01E+06	867.4562	3.4
26	VGVGLPGQR	6442	97	9	441.7643	2	21.2	2.88E+06	881.5082	6.5
27	LLSKKLL	11925	97	7	407.7943	2	40.93	2.04E+06	813.5687	6.5
28	QAGVSPGK	1869	97	8	372.2061	2	7.6	1.97E+06	742.3973	0.5
29	G(+42.01)SPPGEGPAGF	7742	97	11	507.7345	2	25.69	1.73E+06	1013.4454	8.9
30	TGAGEVGKSPDR	1254	97	12	587.2987	2	5.93	8.98E+05	1172.5786	3.6
31	LVQDLM(+15.99)ETDLY	10546	96	11	678.3273	2	36.24	6.47E+06	1354.6326	5.5
32	Q(+42.01)LGADGR	2305	96	7	379.6941	2	8.99	5.25E+06	757.3718	2.5
33	A(+42.01)GAEKEGSSAPA	1255	96	12	558.7515	2	5.98	3.02E+06	1115.5095	-19
34	QGPSGPSEGR	1519	96	10	486.2334	2	6.69	1.82E+06	970.4468	5.6
35	LGDSPGR	2173	96	7	351.1832	2	8.02	9.51E+05	700.3504	2.1
36	A(+42.01)GPGSPVGVGAP	5647	96	12	504.2639	2	18.62	7.37E+05	1006.5083	4.9
37	GSVGPAGAVGRP	4393	96	12	512.7833	2	14.89	4.66E+05	1023.5461	5.8
38	LVHRDLKPSNL	3816	96	11	646.3867	2	13.09	0	1290.7407	14
39	EGDAGAQGPPGPAGPAGER	4480	95	19	845.8934	2	14.89	2.42E+07	1689.7705	1.1
40	M(+15.99)ETDLNEFFVLYDVQHGY	13671	95	18	1118.5183	2	45.87	2.07E+07	2234.9829	17.5
41	AGPPGSDGGAPGAK	1836	95	14	569.7812	2	7.27	1.03E+07	1137.5415	5.7
42	Q(+42.01)FGADGVAGPK	6071	95	11	544.7751	2	20.1	8.87E+06	1087.5298	5.4
43	GPAGPAGPR	1867	95	9	390.2128	2	6.95	6.01E+06	778.4085	3.3
44	SGPAGPR	1206	95	7	321.1724	2	5.85	3.09E+05	640.3292	1.5
45	QKPGAEGKNGN	1574	95	11	550.2806	2	6.66	2.38E+05	1098.5417	4.5
46	G(+42.01)SPPGEGQPA	3329	95	10	469.717	2	11.72	1.45E+05	937.4141	5.8

Figure S1. Peptide identification results (confidence score ≥95)

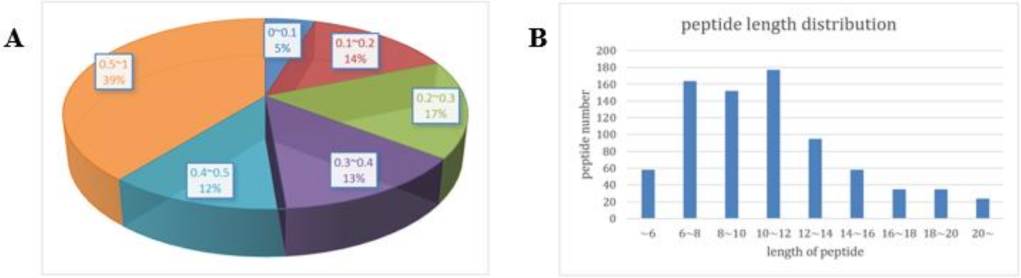

Figure S2. Predicted results of bioactivity(A) and length distribution(B) of identified mulberry leaf peptide

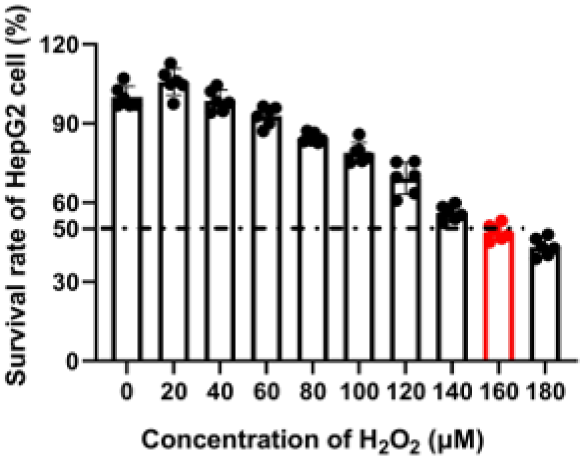

Figure S3. Effects of different concentrations of H_2O_2 on the survival rate of HepG2 cells. The values are expressed as the means ± SD (n=6). HepG2 cells were treated with H_2O_2 (0-180 μM) for 24h, and the survival rate was determined by CCK8.
